# LiNa_5_Mo_9_O_30_

**DOI:** 10.1107/S1600536812041876

**Published:** 2012-10-13

**Authors:** Hamadi Hamza, Ines Ennajeh, Mohamed Faouzi Zid, Ahmed Driss

**Affiliations:** a Laboratoire de Matériaux et Cristallochimie, Faculté des Sciences de Tunis, Université de Tunis ElManar, 2092 Manar II Tunis, Tunisia

## Abstract

The tite compound, lithium penta­sodium nona­molybdate, LiNa_5_Mo_9_O_30_, was synthesized by solid-state reaction. The three-dimensional [Mo_9_O_30_]^6−^ framework is built up from MoO_6_ octa­hedra and MoO_5_ bipyramids, linked together by edges and corners. The framework delimits two types of inter­secting tunnels running along [100] and [010] in which the Na^+^ and Li^+^ ions are located. The asymmetric unit contains one Mo, one Na and one Li site located on a twofold rotation axis. The crystal studied was a racemic twin with site a twin ratio of 0.51 (10):0.49 (10). Relationships between the structures of K_2_Mo_3_O_10_, K_2_Mo_4_O_13_, Cs_2_Mo_7_O_22_, Na_6_Mo_10_O_33_ and Na_6_Mo_11_O_36_ compounds are discussed.

## Related literature

For background to the physico-chemical properties of related compounds, see: Mizushima *et al.* (1980 ▶); Thackeray *et al.* (1984 ▶); Dahn *et al.* (1991 ▶); Tarascon *et al.* (1991 ▶); Kanno *et al.* (1994 ▶); Yuh *et al.* (1995 ▶); Broussely *et al.* (1995 ▶); Capitaine *et al.* (1996 ▶); Delmas *et al.* (1999 ▶); Bruce *et al.* (1999 ▶); Guilmard *et al.* (2003 ▶). For similar structure types, see: Caillet (1967 ▶); Seleborg (1967 ▶); Gatehouse *et al.* (1983 ▶). For background to their electronic properties, see: Aranda *et al.* (1992 ▶); Nguyen & Sleight, (1996 ▶); Daidouh *et al.* (1997 ▶); Ouerfelli *et al.* (2007 ▶). For details of structural relationships between these compounds, see: Gatehouse & Leverett (1968 ▶); Gatehouse & Miskin (1975 ▶); Eda *et al.* (2004 ▶). For the preparation, see: Bramnik & Ehrenberg (2004 ▶). For bond-valence sums, see: Brown & Altermatt (1985 ▶).

## Experimental

### 

#### Crystal data

LiMo_9_Na_5_O_30_
*M* *_r_* = 1465.35
Orthorhombic, 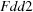

*a* = 7.1927 (8) Å
*b* = 37.159 (4) Å
*c* = 17.925 (2) Å
*V* = 4791.0 (9) Å^3^
*Z* = 8
Mo *K*α radiation
μ = 4.77 mm^−1^
*T* = 298 K
0.30 × 0.20 × 0.10 mm

#### Data collection

Enraf–Nonius CAD-4 diffractometer
Absorption correction: ψ scan (North *et al.*, 1968 ▶) *T*_min_ = 0.31, *T*_max_ = 0.61
3006 measured reflections
2605 independent reflections
2579 reflections with *I* > 2σ(*I*)
*R*_int_ = 0.035
2 standard reflections every 120 min intensity decay: 2.3%

#### Refinement

*R*[*F* ^2^ > 2σ(*F* ^2^)] = 0.035
*wR*(*F* ^2^) = 0.096
*S* = 1.13
2605 reflections
202 parameters
1 restraint
Δρ_max_ = 1.55 e Å^−3^
Δρ_min_ = −1.65 e Å^−3^
Absolute structure: Flack (1983 ▶), 1259 Fridel pairs
Flack parameter: 0.51 (10)

### 

Data collection: *CAD-4 EXPRESS* (Duisenberg, 1992 ▶; Macíček & Yordanov, 1992 ▶); cell refinement: *CAD-4 EXPRESS*; data reduction: *XCAD4* (Harms & Wocadlo, 1995 ▶); program(s) used to solve structure: *SHELXS97* (Sheldrick, 2008 ▶); program(s) used to refine structure: *SHELXL97* (Sheldrick, 2008 ▶); molecular graphics: *DIAMOND* (Brandenburg, 1998 ▶); software used to prepare material for publication: *WinGX* publication routines (Farrugia, 1999 ▶).

## Supplementary Material

Crystal structure: contains datablock(s) I. DOI: 10.1107/S1600536812041876/vn2054sup1.cif

Structure factors: contains datablock(s) I. DOI: 10.1107/S1600536812041876/vn2054Isup2.hkl

Additional supplementary materials:  crystallographic information; 3D view; checkCIF report

## Figures and Tables

**Fig. 1.**
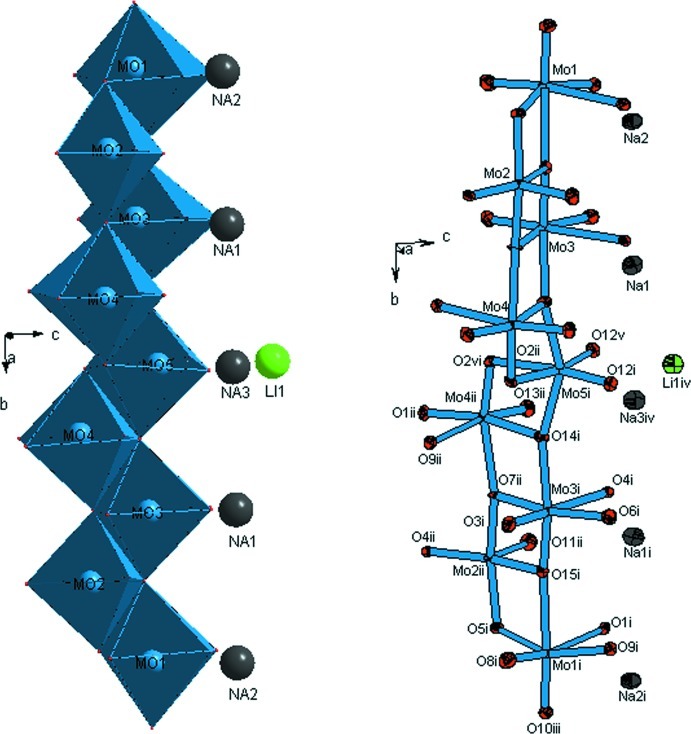
Unité asymétrique dans LiNa5Mo9O30. Les éllipsoïdes ont été définis avec 50% de probabilité. [Code de symétrie: (i) 1/4 + x, 3/4 - y,3/4 + z; (ii) x, 1/2 + y, 1/2 + z; (iii) x - 1/4, 3/4 - y, 1/4 + z; (iv) 1/2 + x, 1/2 + y, 1 + z; (v) 3/4 - x, 1/4 + y, 3/4 + z; (vi) 1 - x, 1/2 - y, 1/2 + z].

**Fig. 2.**
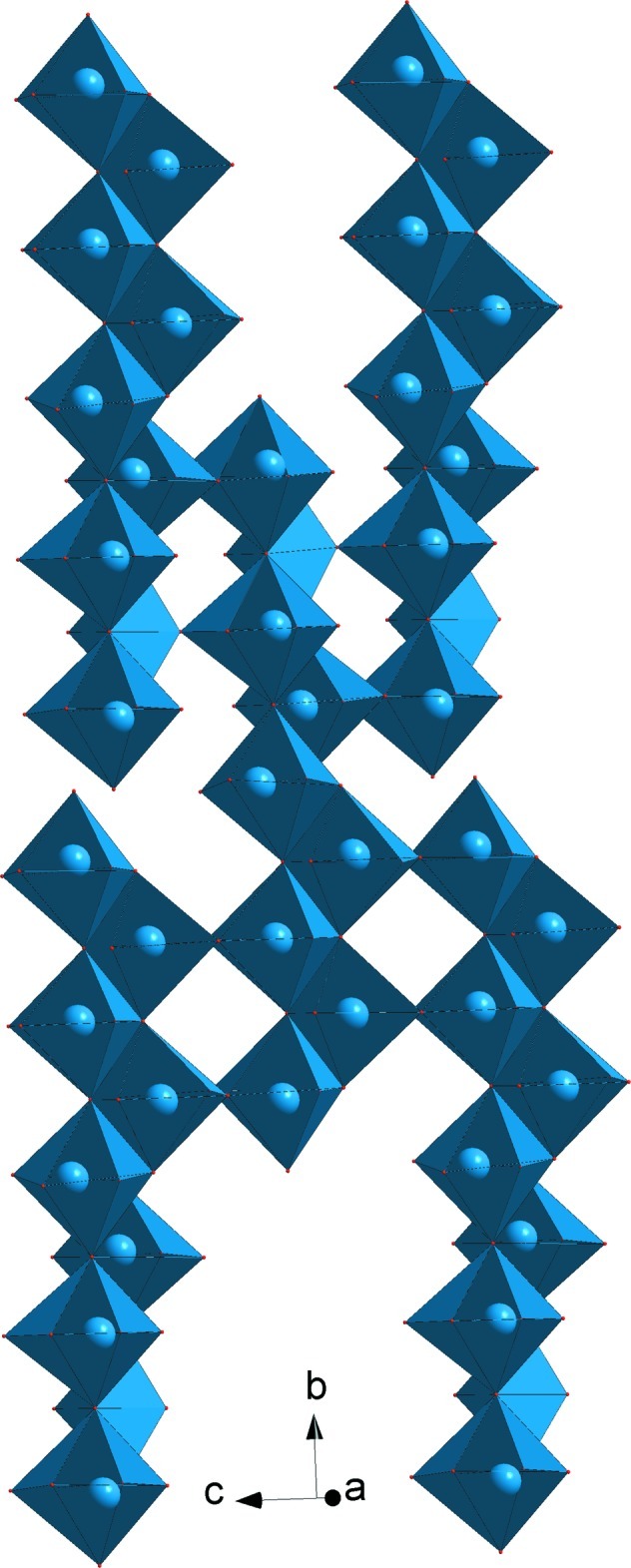
Jonction des unités Mo9O30 dans la charpente anionique de LiNa5Mo9O30, selon a.

**Fig. 3.**
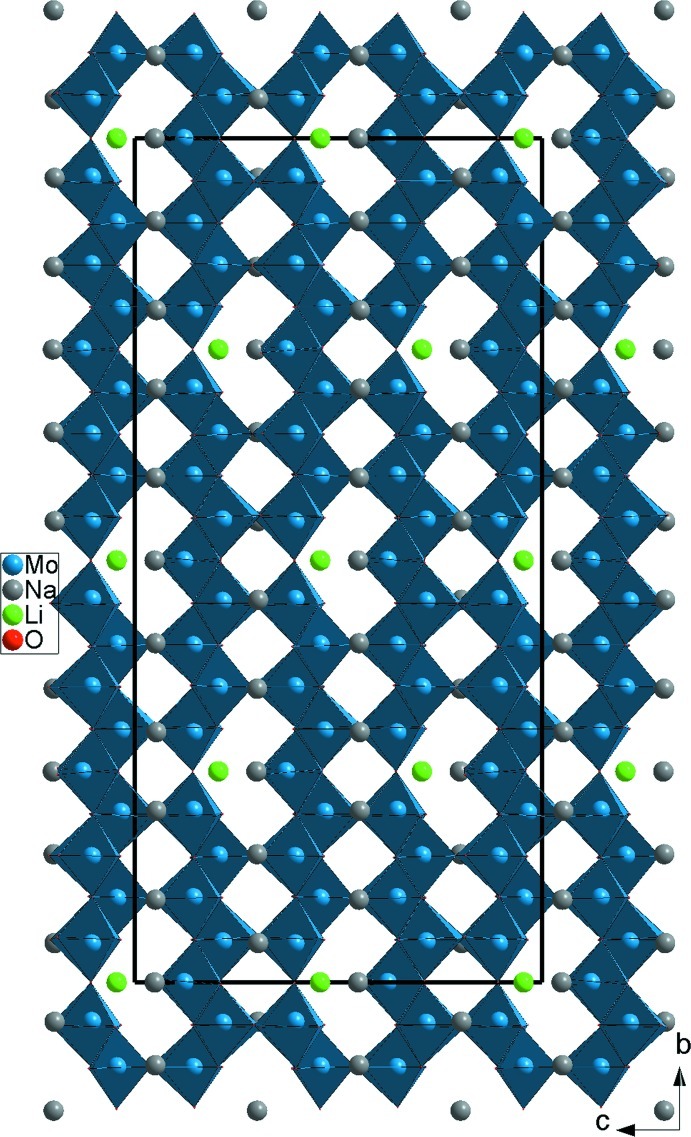
Projection de la structure de LiNa5Mo9O30, selon a, mettant en évidence les canaux où logent les cations Na+.

**Fig. 4.**
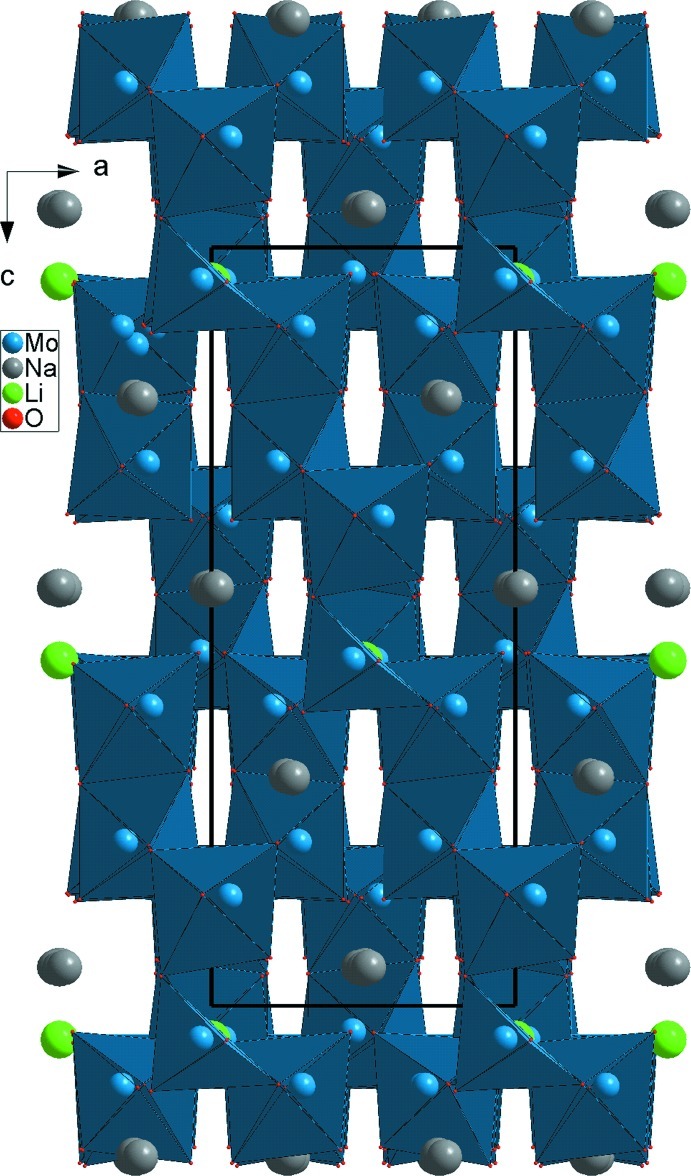
Projection de la structure de LiNa5Mo9O30, selon la direction [010], montrant l'emplacement des cations monovalents.

**Fig. 5.**
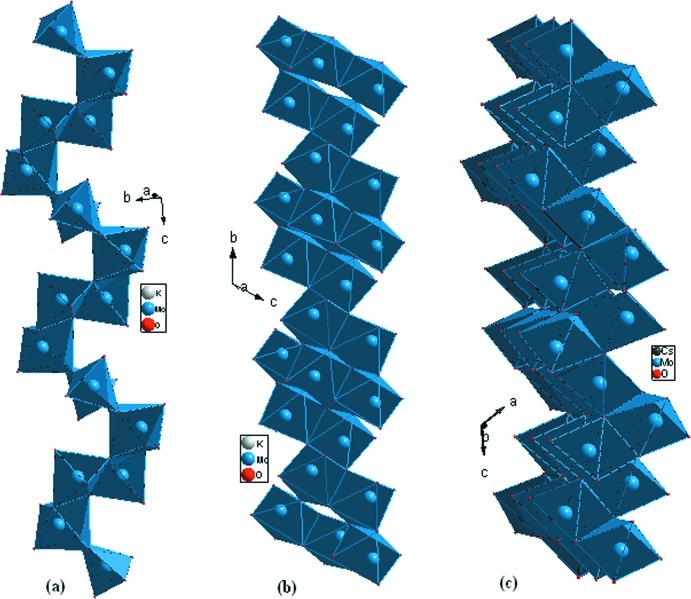
(a) Chaînes ondulées dans K2Mo3O10, (b) Rubans dans K2Mo4O13, (c) Couches dans Cs2Mo7O22.

## supplementary crystallographic information

### Comment

Les matériaux à structure ouverte, en particulier les oxydes mixtes à cations monovalents, constituent un vaste domaine de recherche dans lequel travaille actuellement un grand nombre de laboratoires dans le monde. Ces matériaux présentent des propriétés physiques intéressantes tels que la conductivité ionique (Daidouh *et al.*, 1997), échange d'ions (Aranda *et al.*, 1992), magnétiques (Ouerfelli *et al.*, 2007) ou parfois catalytique (Nguyen & Sleight, 1996). La découverte des batteries de type Li-ion rechargeable tel que les batteries à base de LiCoO_2_ (Yuh *et al.*, 1995) a encouragé la recherche dans cet axe, en raison de leur forte densité énergétique, faible coût des matières premières et respect de l'environnement et de sécurité. Néanmoins, plusieurs travaux s'intéssent à remplacer l'oxyde LiCoO_2_ par d'autres permettant un meilleur fonctionnement de la batterie telques les matériaux Li*_x_*MO_2_ (*M*= Mn, Fe, Co, Ni) (Broussely *et al.*, 1995; Mizushima *et al.*, 1980; Kanno *et al.*, 1994), LiMn_2_O_4_ (Thackeray *et al.*, 1984; Tarascon *et al.*, 1991), LiNiO_2_ (Guilmard *et al.*, 2003; Dahn *et al.*, 1991), LiNi_1 -_*_y_M_y_*O_2_ (*M*=Co, Fe) (Delmas *et al.*, 1999) et LiMnO_2_ (Capitaine *et al.*, 1996; Bruce *et al.*, 1999) qui ont pris un grand intérêt dans la réalisation des générateurs électrochimiques de haute densité d'énergie. Dans ce cadre, on a essayé d'une part, d'explorer le système Li_2_O–Na_2_O–MoO_3_ et d'autre part d'augmenter la mobilité des ions monovalents dans les composés rencontrés dans la littérature Na_6_Mo_11_O_36_ (Bramnik & Ehrenberg, 2004), Na_6_Mo_10_O_33_ (Gatehouse *et al.*, 1983), Na_2_Mo_3_O_10_ et Na_2_Mo_5_O_16_ (Caillet, 1967), Na_2_Mo_2_O_7_ (Seleborg, 1967) en substituant l'ion sodium par le lithium de taille plus faible. Ceci nous a conduit à la synthèse, par réaction à l'état solide, d'un nouveau molybdène oxyde double de sodium et de lithium de formulation LiNa_5_Mo_9_O_30_. *L*'unité *asym*étrique est construite par deux groupements identiques Mo_4_O_17_ reliés par mise en commun d'arêtes à un octaèdre MoO_6_ (Fig. 1). Dans ces derniers clusters Mo_4_O_17_ trois octaèdres MoO_6_ se connectent au moyen d'une bipyramide trigonale MoO_5_ (Fig. 1). En effet, dans la charpente anionique chaque unité structurale Mo_9_O_30_ se lie respectivement à quatre identiques par partage d'une arête et d'un sommet (Fig. 2). Il en résulte une charpente tridimensionnelle possédant des canaux, parallèles à la direction [100] (Fig. 3), où se situent les cations monovalents Li^+^ et Na^+^. La figure 4 montre l'emplacement de ces derniers en face des polyèdres et non en face des fenêtres disposées selon [010]. Les valeurs des charges des ions (BVS) dans la structure ont été calculées moyennant la formule empirique de Brown (Brown & Altermatt, 1985). Le résultat final: Mo1(5.93), Mo2(6.12), Mo3(6.04), Mo4(6.15), Mo5(6.27), Na1(1.21), Na2(1.13), Na3(1.15), et Li1(1.05) confirme bien les degrés d'oxydation des différents ions dans la phase étudiée. Une étude comparative de notre matériau avec des travaux antérieurs révèle une filiation structurale et un lien de parenté à celles de K_2_Mo_3_O_10_ (Eda *et al.*, 2004), K_2_Mo_4_O_13_ (Gatehouse & Leverett, 1968), Cs_2_Mo_7_O_22_ (Gatehouse & Miskin, 1975), Na_6_Mo_10_O_33_ et Na_6_Mo_11_O_36_ (Bramnik & Ehrenberg, 2004) qui peut être considéré comme un dérivé de la structure d'anatase. En effet, les octèdres MoO_6_ et les pyramides MoO_5_ se connectent dans K_2_Mo_3_O_10_ pour former des chaînes ondulées se propageant selon la direction [001] (Fig. 5a). Ils s'associent dans la charpente anionique unidimensionnelle (one-dimensional) de K_2_Mo_4_O_13_ pour conduire à des rubans disposés selon [010] (Fig. 5 b). Une disposition en dents de scie de ces polyèdres dans la charpente anionique bidimensionnelle (two-dimensional) de Cs_2_Mo_7_O_22_ conduit à des couches orientées paralèllement au plan (100) (Fig. 5c). La cohésion entre octèdres MoO_6_ et pyramides MoO_5_ par mise en commun d'arêtes et de sommets peut engendrer de différentes structures à charpente tridimensionnelles (three-dimensional) rencontrées dans les matériaux de formulation Na_6_Mo_10_O_33_, Na_6_Mo_11_O_36_ et aussi dans notre composé LiNa_5_Mo_9_O_30_.

### Experimental

Dans le but de substituer l'ion Na^+^ par Li^+^ dans Na_6_Mo_11_O_36_ (Bramnik & Ehrenberg, 2004), un mélange a été réalisé dans les rapports molaires Li:Na:Mo égaux à 1:5:11 à partir des réactifs solides LiNO_3_ (Fluka, 62575), NaCO_3_ (Fluka, 71350) e t (NH_4_)_2_Mo_4_O_13_ (Fluka, 69858). Il a été finement broyé et préchauffé à l'air à 573 K pendant une nuit. Après refroidissement et broyage, la préparation est portée, proche de la fusion pour favoriser la germination et la croissance des cristaux, à 858 K pendant deux jours. le résidu final est refroidi lentement (5 K/jour) dans un intervalle de 50 degrés puis rapide jusqu'à la température ambiante. Par lavage à l'eau chaude des cristaux de couleur jaunâtre de qualité et de taille suffisante, ont été séparés pour analyse par DRX.

### Refinement

À la fin des premiers cycles d'affinement un examen de la Fourier-différence finale révèle la présence d'un pic d'intensité faible situé à des distances interatomique des atomes d'oxygène correspondant bien au lithium mais ayant une agitation thermique variable. *L*'utilisation d'un facteur thermique isotrope pour l'ion O15 conduit à des ellipsoïdes bien définis. De plus, les densités d'électrons maximum et minimum restants dans la Fourier différence, sont acceptables et sont situées respectivements à 0.82 Å de O11 et à 0.86 Å de Mo2.

### Crystal data

**Table:**

LiMo_9_Na_5_O_30_	*F*(000) = 5408
*M**_r_* = 1465.35	*D*_x_ = 4.063 Mg m^−^^3^
Orthorhombic, *F**d**d*2	Mo *K*α radiation, λ = 0.71073 Å
Hall symbol: F 2 -2d	Cell parameters from 25 reflections
*a* = 7.1927 (8) Å	θ = 10–15°
*b* = 37.159 (4) Å	µ = 4.77 mm^−^^1^
*c* = 17.925 (2) Å	*T* = 298 K
*V* = 4791.0 (9) Å^3^	Prism, yellow
*Z* = 8	0.30 × 0.20 × 0.10 mm

### Data collection

**Table:**

Enraf–Nonius CAD-4 diffractometer	2579 reflections with *I* > 2σ(*I*)
Radiation source: fine-focus sealed tube	*R*_int_ = 0.035
Graphite monochromator	θ_max_ = 27.0°, θ_min_ = 2.2°
ω/2θ scans	*h* = −9→1
Absorption correction: ψ scan (North *et al.*, 1968)	*k* = −1→47
*T*_min_ = 0.31, *T*_max_ = 0.61	*l* = −22→22
3006 measured reflections	2 standard reflections every 120 min
2605 independent reflections	intensity decay: 2.3%

### Refinement

**Table:**

Refinement on *F*^2^	Secondary atom site location: difference Fourier map
Least-squares matrix: full	*w* = 1/[σ^2^(*F*_o_^2^) + (0.064*P*)^2^ + 107.1435*P*] where *P* = (*F*_o_^2^ + 2*F*_c_^2^)/3
*R*[*F*^2^ > 2σ(*F*^2^)] = 0.035	(Δ/σ)_max_ = 0.003
*wR*(*F*^2^) = 0.096	Δρ_max_ = 1.55 e Å^−^^3^
*S* = 1.13	Δρ_min_ = −1.65 e Å^−^^3^
2605 reflections	Extinction correction: *SHELXL*, Fc^*^=kFc[1+0.001xFc^2^λ^3^/sin(2θ)]^-1/4^
202 parameters	Extinction coefficient: 0.000262 (18)
1 restraint	Absolute structure: Flack (1983), 1259 Fridel pairs
Primary atom site location: structure-invariant direct methods	Flack parameter: 0.51 (10)

### Special details

**Table:**

Geometry. All e.s.d.'s (except the e.s.d. in the dihedral angle between two l.s. planes) are estimated using the full covariance matrix. The cell e.s.d.'s are taken into account individually in the estimation of e.s.d.'s in distances, angles and torsion angles; correlations between e.s.d.'s in cell parameters are only used when they are defined by crystal symmetry. An approximate (isotropic) treatment of cell e.s.d.'s is used for estimating e.s.d.'s involving l.s. planes.
Refinement. Refinement of *F*^2^ against ALL reflections. The weighted *R*-factor *wR* and goodness of fit *S* are based on *F*^2^, conventional *R*-factors *R* are based on *F*, with *F* set to zero for negative *F*^2^. The threshold expression of *F*^2^ > σ(*F*^2^) is used only for calculating *R*-factors(gt) *etc*. and is not relevant to the choice of reflections for refinement. *R*-factors based on *F*^2^ are statistically about twice as large as those based on *F*, and *R*- factors based on ALL data will be even larger.

### Fractional atomic coordinates and isotropic or equivalent isotropic displacement parameters (Å^2^)

**Table:**

	*x*	*y*	*z*	*U*_iso_*/*U*_eq_	
Mo1	0.29758 (9)	0.045998 (17)	0.10592 (3)	0.00887 (17)	
Mo2	0.21226 (10)	0.150362 (15)	0.29407 (3)	0.00799 (17)	
Mo3	0.30801 (11)	0.149366 (14)	0.10618 (4)	0.00796 (16)	
Mo4	0.21380 (9)	0.048223 (16)	0.28412 (3)	0.00822 (17)	
Mo5	0.2500	0.2500	0.12906 (7)	0.0084 (2)	
Na1	0.7664 (7)	0.15195 (10)	0.1994 (3)	0.0243 (8)	
Na2	0.7455 (7)	0.04660 (9)	0.1970 (3)	0.0217 (7)	
Na3	0.5000	0.0000	−0.0485 (4)	0.0268 (11)	
Li1	0.0000	0.0000	0.0442 (13)	0.027 (5)	
O1	0.0671 (12)	0.05040 (18)	0.2076 (4)	0.0148 (16)	
O2	0.7058 (9)	−0.00250 (17)	0.2884 (4)	0.0115 (13)	
O3	0.4672 (11)	0.14957 (17)	0.0356 (7)	0.0172 (16)	
O4	0.0839 (12)	0.15022 (16)	0.2111 (4)	0.0136 (14)	
O5	0.0454 (10)	0.05306 (15)	0.0490 (4)	0.0118 (13)	
O6	0.4433 (11)	0.15057 (18)	0.1869 (4)	0.0158 (17)	
O7	0.2996 (10)	0.10005 (14)	0.2950 (4)	0.0120 (14)	
O8	0.4585 (12)	0.05059 (17)	0.0345 (6)	0.0201 (17)	
O9	0.4246 (11)	0.05372 (18)	0.1904 (3)	0.0106 (13)	
O10	0.7429 (11)	0.0003 (2)	0.6076 (5)	0.0194 (14)	
O11	0.0513 (11)	0.14798 (17)	0.3632 (6)	0.0172 (17)	
O12	0.4310 (10)	0.2463 (2)	0.1896 (4)	0.0162 (14)	
O13	0.0668 (12)	0.05040 (16)	0.3598 (5)	0.0169 (14)	
O14	0.2252 (9)	0.19936 (15)	0.1018 (5)	0.0140 (14)	
O15	0.2204 (8)	0.10175 (16)	0.1040 (5)	0.0120 (13)*	

### Atomic displacement parameters (Å^2^)

**Table:**

	*U*^11^	*U*^22^	*U*^33^	*U*^12^	*U*^13^	*U*^23^
Mo1	0.0116 (3)	0.0054 (3)	0.0096 (3)	0.0007 (2)	−0.0008 (2)	−0.0001 (2)
Mo2	0.0108 (3)	0.0041 (3)	0.0090 (3)	0.00039 (19)	−0.0011 (2)	0.0003 (2)
Mo3	0.0109 (3)	0.0035 (3)	0.0095 (3)	−0.0002 (2)	−0.0018 (2)	0.0001 (2)
Mo4	0.0107 (3)	0.0048 (3)	0.0092 (3)	−0.0004 (2)	−0.0006 (2)	0.0001 (2)
Mo5	0.0140 (4)	0.0032 (4)	0.0079 (4)	−0.0019 (3)	0.000	0.000
Na1	0.0185 (18)	0.0236 (17)	0.0309 (19)	−0.0012 (15)	0.0031 (15)	−0.0018 (16)
Na2	0.0168 (16)	0.0188 (15)	0.0294 (18)	0.0012 (14)	0.0002 (14)	−0.0001 (17)
Na3	0.022 (2)	0.025 (3)	0.034 (3)	0.002 (2)	0.000	0.000
Li1	0.032 (12)	0.047 (15)	0.003 (9)	−0.007 (11)	0.000	0.000
O1	0.019 (4)	0.014 (3)	0.011 (3)	0.005 (3)	−0.007 (3)	0.000 (2)
O2	0.019 (3)	0.008 (3)	0.007 (3)	0.002 (2)	−0.001 (2)	0.001 (2)
O3	0.019 (3)	0.017 (3)	0.016 (4)	0.000 (2)	−0.001 (3)	0.004 (2)
O4	0.025 (4)	0.007 (3)	0.009 (3)	0.001 (2)	−0.003 (3)	−0.0006 (18)
O5	0.012 (3)	0.009 (3)	0.015 (3)	−0.001 (2)	−0.001 (3)	0.000 (2)
O6	0.015 (4)	0.016 (3)	0.016 (4)	0.002 (2)	−0.004 (3)	−0.001 (2)
O7	0.019 (3)	0.004 (3)	0.013 (3)	−0.001 (2)	−0.004 (3)	0.0008 (19)
O8	0.020 (3)	0.017 (3)	0.023 (4)	0.001 (3)	0.001 (3)	0.003 (3)
O9	0.009 (3)	0.011 (3)	0.012 (3)	−0.001 (2)	0.000 (2)	−0.001 (2)
O10	0.023 (3)	0.014 (3)	0.021 (3)	0.007 (2)	−0.011 (4)	−0.004 (3)
O11	0.019 (4)	0.020 (3)	0.013 (5)	0.003 (2)	0.006 (3)	−0.002 (3)
O12	0.021 (3)	0.016 (3)	0.012 (3)	0.001 (3)	−0.002 (3)	0.003 (2)
O13	0.018 (3)	0.020 (3)	0.013 (3)	0.000 (2)	0.007 (3)	−0.002 (3)
O14	0.016 (3)	0.006 (3)	0.020 (3)	−0.002 (2)	0.000 (3)	−0.003 (2)

### Geometric parameters (Å, º)

**Table:**

Mo1—O8	1.735 (10)	Mo5—O2^vi^	2.200 (8)
Mo1—O10^i^	1.743 (8)	Mo5—O2^iii^	2.200 (8)
Mo1—O9	1.792 (7)	Na1—O4^vii^	2.294 (9)
Mo1—O5	2.098 (7)	Na1—O6	2.336 (9)
Mo1—O15	2.145 (6)	Na1—O8^ii^	2.369 (9)
Mo1—O1	2.469 (8)	Na1—O13^viii^	2.417 (8)
Mo2—O11	1.698 (10)	Na1—O11^viii^	2.427 (9)
Mo2—O4	1.750 (8)	Na1—O3^ii^	2.486 (11)
Mo2—O5^ii^	1.833 (6)	Na2—O1^vii^	2.326 (10)
Mo2—O7	1.973 (6)	Na2—O9	2.326 (9)
Mo2—O15^ii^	2.147 (7)	Na2—O10^ix^	2.370 (9)
Mo3—O3	1.707 (11)	Na2—O2	2.468 (8)
Mo3—O6	1.744 (7)	Na2—O3^ii^	2.562 (9)
Mo3—O15	1.879 (6)	Na2—O11^viii^	2.581 (10)
Mo3—O14	1.953 (6)	Na3—O12^iii^	2.309 (7)
Mo3—O7^iii^	2.158 (7)	Na3—O12^x^	2.309 (7)
Mo3—O4	2.478 (8)	Na3—O8^iv^	2.416 (10)
Mo4—O13	1.722 (9)	Na3—O8	2.416 (10)
Mo4—O1	1.732 (8)	Na3—O13^xi^	2.537 (9)
Mo4—O2^iv^	1.796 (6)	Na3—O13^xii^	2.537 (9)
Mo4—O7	2.032 (6)	Li1—O5	2.000 (6)
Mo4—O14^ii^	2.239 (7)	Li1—O5^xiii^	2.000 (6)
Mo4—O9	2.271 (7)	Li1—O10^i^	2.084 (15)
Mo5—O12	1.700 (7)	Li1—O10^xii^	2.084 (15)
Mo5—O12^v^	1.700 (7)	Li1—O12^xiv^	2.29 (2)
Mo5—O14^v^	1.952 (6)	Li1—O12^iii^	2.29 (2)
Mo5—O14	1.952 (6)		
			
O8—Mo1—O10^i^	105.0 (4)	O12—Mo5—O2^iii^	87.7 (3)
O8—Mo1—O9	105.6 (4)	O12^v^—Mo5—O2^iii^	169.4 (3)
O10^i^—Mo1—O9	104.9 (3)	O14^v^—Mo5—O2^iii^	73.3 (3)
O8—Mo1—O5	101.8 (4)	O14—Mo5—O2^iii^	85.2 (3)
O10^i^—Mo1—O5	86.3 (3)	O2^vi^—Mo5—O2^iii^	84.8 (4)
O9—Mo1—O5	146.3 (3)	O4^vii^—Na1—O6	177.1 (3)
O8—Mo1—O15	93.8 (3)	O4^vii^—Na1—O8^ii^	97.9 (3)
O10^i^—Mo1—O15	152.0 (3)	O6—Na1—O8^ii^	84.4 (3)
O9—Mo1—O15	89.5 (3)	O4^vii^—Na1—O13^viii^	86.1 (3)
O5—Mo1—O15	69.4 (2)	O6—Na1—O13^viii^	95.8 (3)
O8—Mo1—O1	170.6 (3)	O8^ii^—Na1—O13^viii^	84.8 (2)
O10^i^—Mo1—O1	84.3 (3)	O4^vii^—Na1—O11^viii^	86.2 (3)
O9—Mo1—O1	73.0 (3)	O6—Na1—O11^viii^	91.4 (3)
O5—Mo1—O1	76.7 (3)	O8^ii^—Na1—O11^viii^	175.7 (4)
O15—Mo1—O1	76.9 (2)	O13^viii^—Na1—O11^viii^	97.0 (4)
O11—Mo2—O4	105.0 (4)	O4^vii^—Na1—O3^ii^	93.6 (3)
O11—Mo2—O5^ii^	103.7 (3)	O6—Na1—O3^ii^	84.3 (3)
O4—Mo2—O5^ii^	102.4 (3)	O8^ii^—Na1—O3^ii^	98.5 (4)
O11—Mo2—O7	99.3 (3)	O13^viii^—Na1—O3^ii^	176.7 (4)
O4—Mo2—O7	99.9 (3)	O11^viii^—Na1—O3^ii^	79.7 (2)
O5^ii^—Mo2—O7	142.2 (3)	O1^vii^—Na2—O9	169.8 (3)
O11—Mo2—O15^ii^	102.9 (4)	O1^vii^—Na2—O10^ix^	93.7 (3)
O4—Mo2—O15^ii^	151.8 (3)	O9—Na2—O10^ix^	94.8 (3)
O5^ii^—Mo2—O15^ii^	74.2 (3)	O1^vii^—Na2—O2	96.1 (3)
O7—Mo2—O15^ii^	71.7 (2)	O9—Na2—O2	90.2 (3)
O3—Mo3—O6	103.9 (4)	O10^ix^—Na2—O2	84.8 (2)
O3—Mo3—O15	102.3 (3)	O1^vii^—Na2—O3^ii^	88.9 (3)
O6—Mo3—O15	103.2 (3)	O9—Na2—O3^ii^	82.2 (3)
O3—Mo3—O14	99.8 (3)	O10^ix^—Na2—O3^ii^	175.1 (4)
O6—Mo3—O14	100.3 (3)	O2—Na2—O3^ii^	99.0 (4)
O15—Mo3—O14	142.5 (3)	O1^vii^—Na2—O11^viii^	81.0 (3)
O3—Mo3—O7^iii^	101.6 (4)	O9—Na2—O11^viii^	92.0 (3)
O6—Mo3—O7^iii^	154.4 (3)	O10^ix^—Na2—O11^viii^	100.8 (3)
O15—Mo3—O7^iii^	73.2 (2)	O2—Na2—O11^viii^	173.8 (3)
O14—Mo3—O7^iii^	73.0 (2)	O3^ii^—Na2—O11^viii^	75.5 (2)
O3—Mo3—O4	178.2 (4)	O12^iii^—Na3—O12^x^	169.4 (5)
O6—Mo3—O4	74.5 (3)	O12^iii^—Na3—O8^iv^	103.1 (3)
O15—Mo3—O4	79.0 (3)	O12^x^—Na3—O8^iv^	83.5 (3)
O14—Mo3—O4	79.6 (3)	O12^iii^—Na3—O8	83.5 (3)
O7^iii^—Mo3—O4	80.0 (3)	O12^x^—Na3—O8	103.1 (3)
O13—Mo4—O1	104.3 (4)	O8^iv^—Na3—O8	104.0 (5)
O13—Mo4—O2^iv^	102.0 (3)	O12^iii^—Na3—O13^xi^	94.8 (3)
O1—Mo4—O2^iv^	105.9 (3)	O12^x^—Na3—O13^xi^	78.2 (3)
O13—Mo4—O7	93.8 (3)	O8^iv^—Na3—O13^xi^	161.8 (2)
O1—Mo4—O7	102.5 (3)	O8—Na3—O13^xi^	81.24 (19)
O2^iv^—Mo4—O7	142.6 (3)	O12^iii^—Na3—O13^xii^	78.2 (3)
O13—Mo4—O14^ii^	95.0 (4)	O12^x^—Na3—O13^xii^	94.8 (3)
O1—Mo4—O14^ii^	159.8 (3)	O8^iv^—Na3—O13^xii^	81.24 (19)
O2^iv^—Mo4—O14^ii^	75.2 (3)	O8—Na3—O13^xii^	161.8 (2)
O7—Mo4—O14^ii^	69.8 (2)	O13^xi^—Na3—O13^xii^	99.3 (4)
O13—Mo4—O9	171.1 (3)	O5—Li1—O5^xiii^	175.1 (14)
O1—Mo4—O9	79.5 (3)	O5—Li1—O10^i^	80.5 (5)
O2^iv^—Mo4—O9	84.4 (3)	O5^xiii^—Li1—O10^i^	96.8 (5)
O7—Mo4—O9	77.4 (3)	O5—Li1—O10^xii^	96.8 (5)
O14^ii^—Mo4—O9	80.6 (3)	O5^xiii^—Li1—O10^xii^	80.5 (5)
O12—Mo5—O12^v^	100.7 (5)	O10^i^—Li1—O10^xii^	113.9 (12)
O12—Mo5—O14^v^	99.7 (3)	O5—Li1—O12^xiv^	100.8 (7)
O12^v^—Mo5—O14^v^	98.7 (3)	O5^xiii^—Li1—O12^xiv^	83.2 (5)
O12—Mo5—O14	98.7 (3)	O10^i^—Li1—O12^xiv^	157.6 (9)
O12^v^—Mo5—O14	99.7 (3)	O10^xii^—Li1—O12^xiv^	88.3 (4)
O14^v^—Mo5—O14	151.0 (5)	O5—Li1—O12^iii^	83.2 (5)
O12—Mo5—O2^vi^	169.4 (3)	O5^xiii^—Li1—O12^iii^	100.8 (7)
O12^v^—Mo5—O2^vi^	87.7 (3)	O10^i^—Li1—O12^iii^	88.3 (4)
O14^v^—Mo5—O2^vi^	85.2 (3)	O10^xii^—Li1—O12^iii^	157.6 (9)
O14—Mo5—O2^vi^	73.3 (3)	O12^xiv^—Li1—O12^iii^	69.8 (8)

Symmetry codes: (i) *x*−1/2, *y*, *z*−1/2; (ii) *x*+1/4, −*y*+1/4, *z*+1/4; (iii) *x*−1/4, −*y*+1/4, *z*−1/4; (iv) −*x*+1, −*y*, *z*; (v) −*x*+1/2, −*y*+1/2, *z*; (vi) −*x*+3/4, *y*+1/4, *z*−1/4; (vii) *x*+1, *y*, *z*; (viii) *x*+3/4, −*y*+1/4, *z*−1/4; (ix) −*x*+3/2, −*y*, *z*−1/2; (x) −*x*+5/4, *y*−1/4, *z*−1/4; (xi) *x*+1/2, *y*, *z*−1/2; (xii) −*x*+1/2, −*y*, *z*−1/2; (xiii) −*x*, −*y*, *z*; (xiv) −*x*+1/4, *y*−1/4, *z*−1/4.
